# Monthly Report of Deaths in the City of Buffalo, for the Month of December, 1864

**Published:** 1865-01

**Authors:** Sandford Eastman

**Affiliations:** Health Physician


*Monthly Report of Deaths in the City of Buffalo, for the month of December*, 1864.

Whole number of deaths from disease, 132. In addition to the above, 4 still-born were reported in the city.

LOCALITY.—City at large, 113; Hospital of Sisters of Charity, 3; Buffalo General Hospital, 2; Catholic Foundling Asylum, 6; Erie County Alms House, 8; Small-Pox Hospital, 2.

BY WHOM CERTIFIED.—By regular Physicians at Public Institutions, 23; by regular Physicians in city at large, 74; by irregular Practitioners, 16; by Coroner, 6; by Undertakers, 17.

CAUSES OF DEATH.—Accident, 2; accident by drowning, 1; albuminuria, 1; apoplexy cerebri, 1; bronchitis, 1; cancer, 1; cancer of the stomach, 2; cholera morbus, 1; consumption, 20; convulsions, 6; croup, 7; croup diptheritic, 5; debility, 1; dentition, 1; diarrhœa, 4; disease of the heart, 4; disease of the kidneys, 1; diptheria, 4; dropsy general, 3; dysentery, 1; Erysipelas, 3; fever, 1; fever puerperal, 3; scarlet fever, 1; typhoid fever, 11; typhus fever, 1; hæmorrhage from lungs, 1; inanition, 5; inflammation of bowels, 2; inflammation of brain and meninges, 3; inflammation of lungs, 7; inflammation of lungs, typhoid, 5; inflammatiou of lungs and pleura, 1; inflammation of stomach, 2; Bright's disease of kidneys, 1; measles, 1; old age, 3; parturition, 1; premature birth 1; pyæmia, 2; small-pox, 7; syphilis 1; Tetanus, 1; unknown, 1.

The number of deaths in the present year is 71 less than in the last year, and 16 less than the average for five years.

SANDFORD EASTMAN, M. D., Health Physician.

